# Cohort profile: biological pathways of risk and resilience in Syrian refugee children (BIOPATH)

**DOI:** 10.1007/s00127-022-02228-8

**Published:** 2022-01-18

**Authors:** Fiona S. McEwen, Cassandra Popham, Patricia Moghames, Demelza Smeeth, Bernadette de Villiers, Dahlia Saab, Georges Karam, John Fayyad, Elie Karam, Michael Pluess

**Affiliations:** 1 Biological and Experimental Psychology, School of Biological and Behavioural Sciences, Queen Mary University of London, London, UK; 2 Médecins du Monde, Beirut, Lebanon; 3 Institute for Development, Research, Advocacy and Applied Care, Beirut, Lebanon; 4 Saint George Hospital University Medical Center, Beirut, Lebanon; 5 Faculty of Medicine, Balamand University, El-Koura, Lebanon

**Keywords:** Cohort study, Syrian refugees, War exposure, Displacement, Child and adolescent mental health, Resilience

## Abstract

The BIOPATH cohort was established to explore the interplay of psychosocial and biological factors in the development of resilience and mental health problems in Syrian refugee children. Based in Lebanon, a middle-income country significantly impacted by the refugee crisis, it is the first such cohort of refugees in the Middle East. Families were recruited from informal tented settlements in the Beqaa region using purposive cluster sampling. At baseline (October 2017–January 2018), *N* = 3188 individuals participated [*n* = 1594 child–caregiver dyads; child gender, 52.6% female; mean (SD) age = 11.44 (2.44) years, range = 6–19]. Re-participation rate at 1-year follow-up was 62.8%. Individual interviews were conducted with children and primary caregivers and biological samples collected from children. Measures include: (1) children’s well-being and mental health problems (using tools validated against clinical interviews in a subsample of the cohort); (2) psychosocial risk and protective factors at the level of the individual (e.g. coping strategies), family (e.g. parent–child relationship), community (e.g. collective efficacy), and wider context (e.g. services); (3) saliva samples for genetic and epigenetic (methylation) analyses; (4) hair samples to measure cortisol, dehydroepiandrosterone (DHEA) and testosterone. This cohort profile provides details about sampling and recruitment, data collection and measures, demographic data, attrition and potential bias, key findings on resilience and mental health problems in children and strengths and limitations of the cohort. Researchers interested in accessing data should contact Professor Michael Pluess at Queen Mary University of London, UK (e-mail: m.pluess@qmul.ac.uk).

**Supplementary Information:**

The online version contains supplementary material available at 10.1007/s00127-022-02228-8.

The crisis in Syria precipitated by the civil war has led to the displacement of more than 5 million Syrians into surrounding countries, almost half of whom are children and adolescents [1]. As well as having experienced traumatic war-related events, many children and adolescents displaced in low- and middle-income countries (LMICs) face ongoing adversities including insufficient access to basic resources, unstable and poor quality accommodation, and limited access to education. Despite the majority of refugees globally living in LMICs, and one in three living in camps, much research to date has been conducted with refugees who have moved to high-income countries [2]. It is particularly important to establish cohorts in LMICs to address this research gap. This cohort comprised Syrian refugees living in informal tented settlements (ITS) in Lebanon. The cohort was established in 2017 and funded by the Eunice Kennedy Shriver National Institute of Child Health and Human Development (Grant number: R01HD083387).

Children exposed to war and adversity are at increased risk of developing mental health problems [3, 4], but there is substantial variation in how children respond and some show remarkable resilience [4, 5]. Variation in how children respond to adversity is likely to be influenced by multiple factors at individual, family, and community levels; however, it is rare for factors across different levels to be measured in the same samples and over time [2]. Factors known to be important in long-term development, like school and peer relationships, are less often studied than individual-level factors that are easier to measure [2]. Furthermore, few studies have focused on the biological embedding of adverse experiences in refugee children: how experiences “get under the skin”. Disruption to neuroendocrine systems, such as cortisol dysregulation, may be a way in which adverse experiences have longer-term consequences on mental or physical health. However, whether cortisol has utility as an unbiased biological signature of previous adversity or is predictive of mental health problems is not yet clear [6]. Genetic and epigenetic factors may also play a role in the response to adverse experiences. For example, a genome-wide polygenic indicator of environmental sensitivity has recently been shown to interact with exposure to stressful life events in predicting depression [7]. This cohort study has the overall aim of exploring the interplay between (potentially modifiable) psychosocial factors and biological factors in the development of risk and resilience in Syrian refugee children. Firstly, we aim to investigate how many children can be considered resilient, which factors predict risk or resilience to war exposure and displacement, and how resilience changes over time. Secondly, we aim to estimate the prevalence and predictors of mental health problems in children. To facilitate this, we have collected data to establish the reliability, validity, and optimal cutoffs for measures of mental health problems when used in this specific population and context. Thirdly, we aim to investigate the role of biological factors in the response to trauma, and specifically neuroendocrine and genome-wide genetic and epigenetic factors. Finally, we aim to explore the interplay between those psychosocial and biological factors found to be most important in predicting risk and resilience.

## Recruitment and follow-up

The study employs a longitudinal cohort design with baseline data assessment and follow-up 1 year later. Syrian refugee children and adolescents reported to be aged 8–16 years at baseline, along with their primary caregiver, were visited and interviewed in the community. Various mental health outcomes were measured, as well as a range of potential risk and resilience factors. Saliva samples were collected for DNA extraction and hair samples were collected for neuroendocrine analyses. A structured clinical interview was conducted with a subsample of families to provide data on the validity of the mental health outcome measures in this sample. The study was granted ethical approval by a local Institutional Review Board and the Ministry of Public Health (Supplementary Section 1).

Participants were families displaced by the Syrian civil war that began in March 2011 and were living as refugees in ITSs in the West and Central Beqaa regions of Lebanon. Purposive cluster sampling was used, selecting seven municipalities with varying levels of vulnerability. In these localities, small- to medium-sized ITSs were selected. In each ITS, all present families were approached. Eligibility criteria were: (1) family who left Syria because of the war within the 4 years prior to recruitment; (2) child aged 8–16 years old; and (3) primary caregiver available to participate (typically the mother, or the caregiver who spent most time with the child). Where more than one child in the family fell into the age range, the child whose birthday was closest to the date of recruitment was selected. Families who were interested and eligible completed the informed consent procedure and baseline data collection was then completed with the child and caregiver from each family. Follow-up was completed 1 year later. See Supplementary Section 2 for details about sampling and recruitment.

**Fig. 1:**
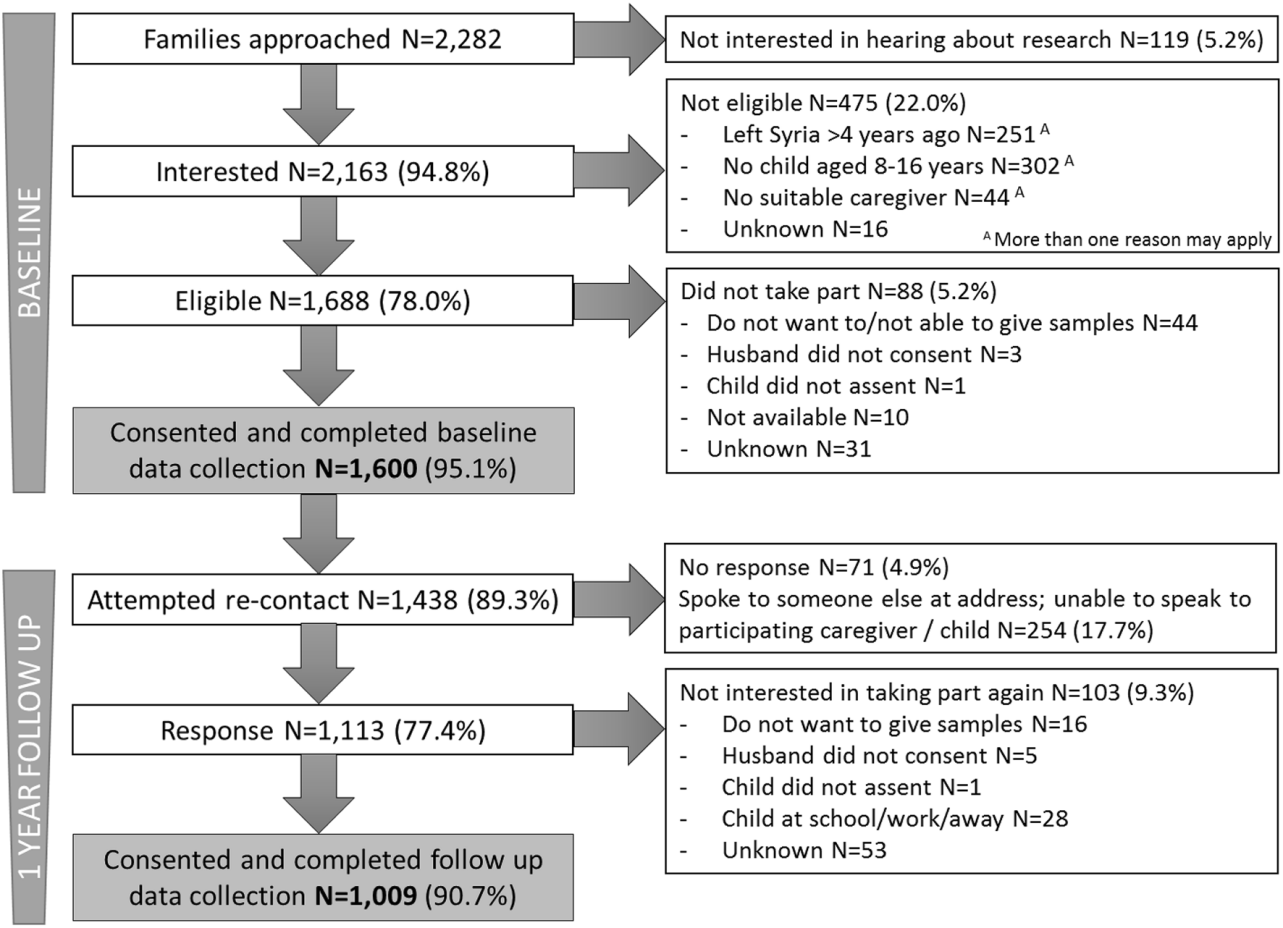
BIOPATH recruitment and follow-up. Data from *n* = 6 families were excluded at baseline, giving final *n* = 1594; data from *n* = 8 families were excluded at follow-up, giving final *n* = 1001

The numbers for recruitment and follow-up are shown in Fig. 1. At baseline, 77 ITSs were visited and *N* = 2282 families approached, *n* = 1600 (70.1%) of whom were eligible, consented to participate, and completed data collection. Given the known mobility of this population, we expected to be able to follow-up approximately two-thirds of the initial sample. Consistent with our predictions, we were able to contact or get information about *n* = 1438 families across 70 ITSs, *n* = 1009 of whom consented to participate and completed data collection (re-participation rate 63%). Some ITSs from baseline were not visited or were visited, but not all families interviewed because residents were working, bad weather prevented visits, or the study period had ended. Of the families the team re-contacted or got information about, *n* = 311 (21.6%) had relocated, the majority (*n* = 222, 71.4%) of whom it was known where they had moved to. Over a quarter (*n* = 64, 28.8%) were reported to have moved back to Syria or to another country. It was possible to follow up *n* = 17 families who had moved within the region where data collection was taking place; the majority of families followed up (*n* = 992, 98.3%) were still living in the same location. Data from six families were completely excluded at baseline and from eight families at follow-up, giving a final sample size of *n* = 1594 dyads at baseline and *n* = 1001 at follow-up (Supplementary Section 2).

After the 1-year follow-up, *n* = 145 families were approached and invited to complete a follow-up clinical interview, *n* = 101 (69.7%) of whom consented to participate and completed data collection. Data from a further *n* = 33 families who had been recruited from BIOPATH families to a linked clinical trial were included, giving a final sample of *n* = 134 with clinical interview data (Figure S1). Families were selected to be representative of the cohort in terms of (1) the proportion who had reported that their child had a need for mental health services, and (2) the proportion with elevated scores on self-reported and parent-reported mental health questionnaires (Figure S1).

## Description of the cohort

Descriptive data for the sample at baseline and follow-up are shown in Table 1. Approximately, half of children were female and the actual age range was 6–19 years at baseline (due to uncertainty about birth dates, some children outside the intended age range were recruited; see Supplementary Section 3). The majority of caregivers who took part were mothers. At follow-up, the same caregiver was available in *n* = 926 cases (92.5%); otherwise a different caregiver took part (*n* = 65, 6.5%) or the child took part without a caregiver (*n* = 9, 0.9%). Of the subsample who completed a clinical interview, *n* = 63 (47.0%) children were female and mean age was 12.15 years (SD = 2.15, range 8–19); *n* = 130 (97.0%) mothers or other female caregivers and *n* = 20 (14.9%) fathers attended the appointment (in *n* = 16 cases, both parents attended). The mean age of the primary caregiver was 38.07 (SD = 7.54, range 16–57). Further details about this subsample have been reported elsewhere [8, 9].

**Table 1:** Description of the cohort

	Baseline (*N* = 1594)	1-Year follow-up (*N* = 1001)	Comparison of those retained vs. not retained	
			Test statistics	Details
Child gender, *N* (%) female	839 (52.6%)	536 (53.5%)	*χ*^2^ (1) = 0.90, *p* = 0.344, tau^C^ = 0.001	
Child age at assessment, mean (SD) [range]^A^	11.44 (2.44) [6–19]	12.25 (2.38) [8–20]	*t* (1191.32) = 3.85, *p* < 0.001, d = 0.20	Retained children are younger than those not retained
Caregiver gender, *N* (%) female	1520 (95.4%)	967 (97.6%)	*χ*^2^ (1) = 3.39, *p* = 0.066, tau^C^ = 0.002	
Caregiver age at assessment, mean (SD) [range] ^A^	39.03 (8.59) [18–75]	39.59 (8.42) [15–76]	t (1589) = 1.48, *p* = 0.139, d = 0.08	
Caregiver relationship to child, *N* (%)				
Mother	1424 (89.3%)	907 (90.6%)	*χ*^2^ (11) = 30.02, *p* = 0.002, tau^C^ = 0.02	Included families more likely to have mother as caregiver
Father	65 (4.1%)	24 (2.4%)		
Stepmother	25 (1.6%)	21 (2.1%)		
Grandmother	24 (1.5%)	14 (1.4%)		
Sister	21 (1.3%)	7 (0.7%)		
Aunt	18 (1.1%)	8 (0.8%)		
Brother	6 (0.4%)	0 (0.0%)		
Uncle	3 (0.2%)	0 (0.0%)		
Cousin	3 (0.2%)	1 (0.1%)		
Other	5 (0.3%)	8 (0.8%)		
Missing ^B^	0 (0.0%)	11 (1.1%)		
Time since leaving Syria, *N* (%)				
≤ 3 years ago (baseline)/≤ 4 years ago (follow-up)	744 (46.7%)	465 (46.5%)	*χ*^2^ (1) = 22.95, *p* < 0.001, *d*^D^ = 0.12	Included families more likely to have left Syria more than 3 years ago
> 3 years ago (baseline)/> 4 years ago (follow-up)	844 (52.9%)	536 (53.5%)		
Missing	6 (0.4%)	0 (0.0%)		
Child nationality, *N* (%)				
Syrian	1571 (98.6%)	988 (98.7%)	*χ*^2^ (4) = 5.25, *p* = 0.263, tau^C^ = 0.003	
Lebanese	8 (0.5%)	3 (0.3%)		
Palestinian	13 (0.8%)	9 (0.9%)		
Iraqi	1 (0.1%)	1 (0.1%)		
Missing	1 (0.1%)	0 (0.0%)		
Caregiver nationality, *N* (%)				
Syrian	1577 (98.9%)	980 (97.9%)	*χ*^2^ (3) = 6.12, *p* = 0.106, tau^C^ = 0.003	
Lebanese	8 (0.5%)	4 (0.4%)		
Palestinian	5 (0.3%)	5 (0.5%)		
Iraqi	1 (0.1%)	1 (0.1%)		
Missing ^B^	3 (0.2%)	11 (1.1%)		
Child married/engaged, *N* (%)	26 (1.6%)	29 (2.9%)	*χ*^2^ (1) = 0.08, *p* = 0.776, tau^C^ = 0.00	
UNHCR vulnerability rating, *N* (%)				
Most vulnerable	615 (38.6%)	342 (34.0%)	*χ*^2^ (2) = 30.03, *p* < 0.001, *d*^D^ = 0.08	Included families less likely to be from most vulnerable localities
Second most vulnerable	648 (40.6%)	454 (45.1%)		
Third most vulnerable	332 (20.8%)	209 (20.8%)		
Missing	0 (0.0%)	2 (0.2%)		
Family members registered with UNHCR, *N* (%)				
All	1253 (78.6%)	866 (86.5%)	*χ*^2^ (2) = 17.42, *p* < 0.001, *d*^D^ = − 0.09	Included families more likely to be registered with UNHCR
Some	174 (10.9%)	99 (9.9%)		
None	163 (10.2%)	18 (1.8%)		
Missing ^B^	4 (0.3%)	18 (1.8%)		
Number of people in the household, median (IQR) [range]				
Adults	2 (1) [1–11]	2 (1) [1–12]	*U* = 290,467.5, *p* = 0.835	
Children	5 (2) [1–18]	5 (2) [1–16]	*U* = 286,022.0, *p* = 0.353	
Total	7 (3) [2–24]	7 (3) [2–22]	*U* = 283,132.5, *p* = 0.361	
Caregiver has current job, *N* (%)				
No	1384 (86.8%)	817 (81.6%)	*χ*^2^ (1) = 6.45, *p* = 0.011, tau^C^ = 0.004	Included families were less likely to report the primary caregiver having a job
Yes	206 (12.9%)	174 (17.4%)		
Wholesale and retail	1 (0.1%)	2 (0.2%)		
Other services, e.g. hotel, transport, cleaning, childcare	10 (0.6%)	18 (1.8%)		
Agriculture	169 (10.6%)	137 (13.7%)		
Construction	1 (0.1%)	0 (0.0%)		
Manufacturing	6 (0.4%)	0 (0.0%)		
Other	14 (0.9%)	16 (1.6%)		
Missing	4 (0.3%)	10 (1.0%)		
Child has access to education, *N* (%)				
No school	621 (39.0%)	542 (54.1%)	*χ*^2^ (2) = 20.79, *p* < 0.001, *d*^D^ = 0.09	Included children were more likely to have access to education
Some education	398 (25.0%)	209 (20.9%)		
School	573 (35.9%)	249 (24.9%)		
Missing	2 (0.1%)	1 (0.1%)		

^A^Age is the best estimate rounded to the nearest year, based on all date of birth and age data available (see Supplementary Section 1); caregiver age is missing for *n* = 3 cases

^B^At follow-up, *n* = 9 children took part without a caregiver and for *n* = 1 caregiver data were missing due to tablet failure; caregiver reported data were missing for *n* = 10 cases

^C^Goodman–Kruskal tau

^D^Somers’ *d*

The vast majority of children and caregivers were of Syrian nationality and most caregivers reported being Sunni Muslim (*n* = 1543, 96.8%). Just under half had left Syria in the past 3 years and the remainder left more than 3 years prior to recruitment. In the majority of cases, some or all family members were registered with UNHCR, the UN Refugee Agency (89.5% at baseline and 96.3% at follow-up). At baseline, 61.0% of children had access to at least some education, reducing to 45.6% by follow-up. Adult literacy was limited—able to read and write only a little or not at all—in over half of the households (*n* = 905, 56.7%). Over half (*n* = 901, 56.5%) of the caregivers reported that they had not attended school and in over half (*n* = 870, 54.5%) of the families the highest earner had previously worked in elementary occupations. Asked at follow-up, over a fifth (*n* = 218, 21.8%) of caregivers reported that the child’s father had more than one wife (range 1–5), and over half (*n* = 535, 53.5%) reported that the child’s parents were related (typically first or second cousins). See Table 1 and Supplementary Sections 4–5 for further description of the sample.

## Frequency of follow-up and attrition

Recruitment and baseline data collection were completed between October 2017 and January 2018. Follow-up was completed 1 year later, between October 2018 and January 2019 [mean (SD) = 51.55 (1.84) weeks]. The subsample that completed a clinical interview was recruited and interviewed between December 2018 and August 2019 [mean (SD) = 27.02 (7.16) weeks later]. No further follow-up assessments were planned due to the high mobility of the population.

Those who participated at follow-up did not differ from those who did not participate in terms of child or caregiver gender, caregiver age, nationality, child marriage, household size, caregiver education or pre-war employment. Of families who re-participated, children were slightly younger than those who did not participate, more likely to participate with their mother, to have left Syria more than 3 years before recruitment, to be registered with UNHCR, and to have access to education. They were less likely to be from the most vulnerable localities and caregivers were less likely to be working or have very low literacy levels. However, all these differences were small (Table 1 and Supplementary Section 5). The subsample who completed a clinical interview did not differ from the remaining sample in terms of child or caregiver gender, time since leaving Syria, child marriage, UNHCR registration, or caregiver education, literacy or employment. Children who completed a clinical interview were more likely to be from the most vulnerable localities, were slightly younger and had younger caregivers, had fewer adults in the household, and were more likely to have access to education than those who did not participate. Other than the bias towards more vulnerable localities, the differences were small (Supplementary Section 6).

For psychosocial measures, there were relatively little missing data other than for measures relating to fathers/male caregivers (because a significant minority of children reported that their father did not live with them) and measures about school (approx. 40% of children did not have access to any education). Missing data for scales was mostly limited to one to two items [8].

## Measures

Children and caregivers were interviewed separately using questionnaires translated into Arabic (see [8] for translation protocol). All questionnaires were piloted in the same population and, where necessary, modified to ensure comprehensibility and good performance (e.g. maintaining good internal consistency and factor structure in scales that were shortened). Visual aids were used to support verbal use of the Likert scale response format (Figure S2). Questionnaires covered both positive (e.g. well-being, optimism [10, 11]) and negative (e.g. depression, PTSD, externalising behaviour problems [12–19]) outcomes. War exposure was measured using a 25-item checklist of war events [20] and both children and caregivers reported on the child’s war exposure. A range of factors with potential risk or protective functions were covered, including those at the individual level (e.g. the temperament trait of environmental sensitivity, self-esteem [21, 22]), the family and community environment (e.g. caregiver–child relationship, caregiver mental health, bullying, social support, school environment [23–29]), and the wider context (e.g. access to services). The subsample selected for clinical interview completed the MINI KID 6.0 [30]; additional information was gathered where appropriate and a Clinical Global Impression severity (CGI-s) score was assigned [31], with psychiatric diagnoses agreed by consensus during supervision with an experienced clinical psychologist [8, 9]. Domains of measurement are described in Table 2 and Supplementary Section 7.

**Table 2:** Measures

	Baseline *n* = 1594	Follow-up *n* = 1001	Clinical interview subsample *n* = 134
	Oct 2017–Jan 2018	Oct 2018–Jan 2019	Dec 2018–Aug 2019
Demographic data, medical data, puberty, mental health service use and needs	X	X	
Child mental health and well-being: well-being, PTSD, depression, anxiety, externalising behaviour problems, sleep problems, impairment	X	X	X
Child psychopathology: structured neuropsychiatric interview, clinical global impression severity score, consensus diagnosis			X
Individual level factors: optimism, self-esteem, future orientation, self-efficacy, coping strategies, environmental sensitivity, child religiosity	X	X	
Family level factors: child maltreatment, forced marriage, caregiver–child relationship (psychological control, acceptance, parental monitoring, conflict, positive home experiences), caregiver mental health and well-being (well-being, general mental health, PTSD, depression, anxiety, impulsivity, perceived stress), caregiver self-efficacy, caregiver environmental sensitivity, life events, livelihood, basic needs, housing, family environment, learning environment	X	X	
Community level factors and wider context: war exposure, bullying, forced work, child maltreatment outside the home, loneliness and social isolation, perceived social support, perceived security, access to education, school environment (peer victimisation, peer support, teacher support, school connectedness), access to services, community environment, working situation, future mobility, perceived refugee environment, human insecurity, collective efficacy	X	X	
Biometric data: height, weight, waist and hip circumference	X	X	
DNA from saliva: genome-wide genotyping (Illumina Infinium Global Screening Array), genome-wide methylation (Illumina Infinium MethylationEPIC Array)	X	X	
Neuroendocrine measures from hair: cortisol, dehydroepiandrosterone, testosterone	X	X	

See Supplementary Section 5 for full list of items, references, and details about modifications to measures

Children and caregivers were interviewed simultaneously, but separately by different interviewers. Where possible, interviews were conducted in different rooms to try to ensure privacy. Where families were living in shelters that had only one room, the child and caregiver were interviewed at the opposite ends of the room, with their backs to each other, and the interviewers spoke in low voices to try to improve privacy. The use of visual aids that participants could point at to respond provided an alternative response format for children or caregivers who did not want to answer ‘out loud’. The procedure for the MINI Kid interview used in the subsample differed, as this interview is designed to be administered to children ≤ 12 years with their parent or caregiver. In this case, questions about symptoms of mental health problems were directed to children but the caregiver asked for further information if necessary. Children older than 12 years were typically interviewed without their caregiver, but further information was sought from caregivers after the interview for areas where the child’s reporting may have been incomplete (e.g. conduct problems, which may be under-reported by children). The decision about whether to interview the child separately or together with the caregiver(s) was made jointly with each family that completed the MINI Kid. In *n* = 94 (70.1%) families, caregiver and child were interviewed together and in *n* = 40 (29.9%) the interview was completed primarily with the child.

Saliva samples were collected from children at baseline and 1-year follow-up using the Isohelix GeneFix saliva collection device. Genomic DNA was extracted from saliva samples for genome-wide single nucleotide polymorphism genotyping and DNA methylation (epigenetic) analyses (Illumina Infinium Global Screening Array and Illumina Infinium MethylationEPIC Array, respectively). Hair samples were taken from each child at baseline and at 1-year follow-up (if the child’s hair was a minimum of 1 cm in length). The 2 cm of hair closest to the scalp, representing hormone secretion over the previous 2 months, was used to measure cortisol, dehydroepiandrosterone (DHEA) and testosterone levels using commercial ELISAs. See Table 3 for the numbers with samples.

**Table 3:** Biological measures from children

Measure	Description	*N* with data at baseline^A^	*N* with data at 1-year follow-up^A^
Pre-screening	For saliva sample: when last ate, brushed teeth, dental work	1590	985
	For hair sample: frequency of hair washing, use of hair dye/henna, etc.	1592	999
Biometric	Height, weight, waist and hip circumference^B^	912^C^	978
DNA from saliva^D^	Genome-wide genotyping (Illumina Infinium Global Screening Array)	1460	945
	Genome-wide methylation (Illumina Infinium MethylationEPIC Array)	1533	947
Neuroendocrine measures from hair^D^	Cortisol (ELISA)	1589	934
	Dehydroepiandrosterone (ELISA)	1589	934
	Testosterone (ELISA)	1589	934

^A^Number of children with data after data cleaning

^B^Number with a minimum of both height and weight measurement for whom it is possible to calculate BMI

^C^Biometric data were not collected during the first 5 weeks of data collection resulting in missing data for approx. 40% of the baseline sample

^D^Number of samples with data which were sent for each analysis; this work is ongoing therefore the final number of samples for each assay may differ once quality control has been completed

## Key findings to date

Point prevalence of depressive disorder, anxiety disorders, PTSD, and externalising behaviour disorders were estimated in the subsample of children with clinical interview data and in the full cohort. Clinical interview data were used to estimate the optimal cutoff for each psychopathology scale as well as the sensitivity, specificity, positive predictive value (PPV) and negative predictive value (NPV) at that cutoff [8]. Prevalence estimates indicated significantly elevated rates of mental health problems in children, whether based on clinical interview in the subsample (20.1% for depressive disorder, 47.8% for anxiety disorders, 39.6% for PTSD, and 26.9% for conduct/oppositional defiant disorder) or using questionnaire measures (adjusted for the rate of false positives and false negatives) in the whole cohort at baseline (19.0% for depression, 54.3% for anxiety, 36.1% for PTSD, and 27.6% for externalising behaviour problems). There was substantial comorbidity, with 32.1% of children meeting criteria for PTSD and one or more other disorders. The perceived quality of the refugee environment significantly predicted children’s symptoms of depression, PTSD, and externalising behaviour problems and this effect was an order of magnitude greater than other predictors, including exposure to war events, child maltreatment, caregiver-child conflict, and caregiver mental health problems. The importance of the environment—such as the quality of housing, access to services, and the social environment—suggests that scaling up of mental health services as well as the development of policies that address social determinants of mental health will be necessary to address the high burden of mental health problems in this population. Results are discussed in McEwen et al. (in preparation).

There were challenges associated with assessing mental disorders in this population given high levels of current adversity. These challenges, and the approaches taken to improve the cultural and contextual sensitivity of the assessment process, are discussed at length in Kyrillos et al. [9]. Importantly, the performance of the anxiety measure proved not optimal [8] and was excluded for individual level prediction for the resilience analyses discussed below.

**Fig. 2:**
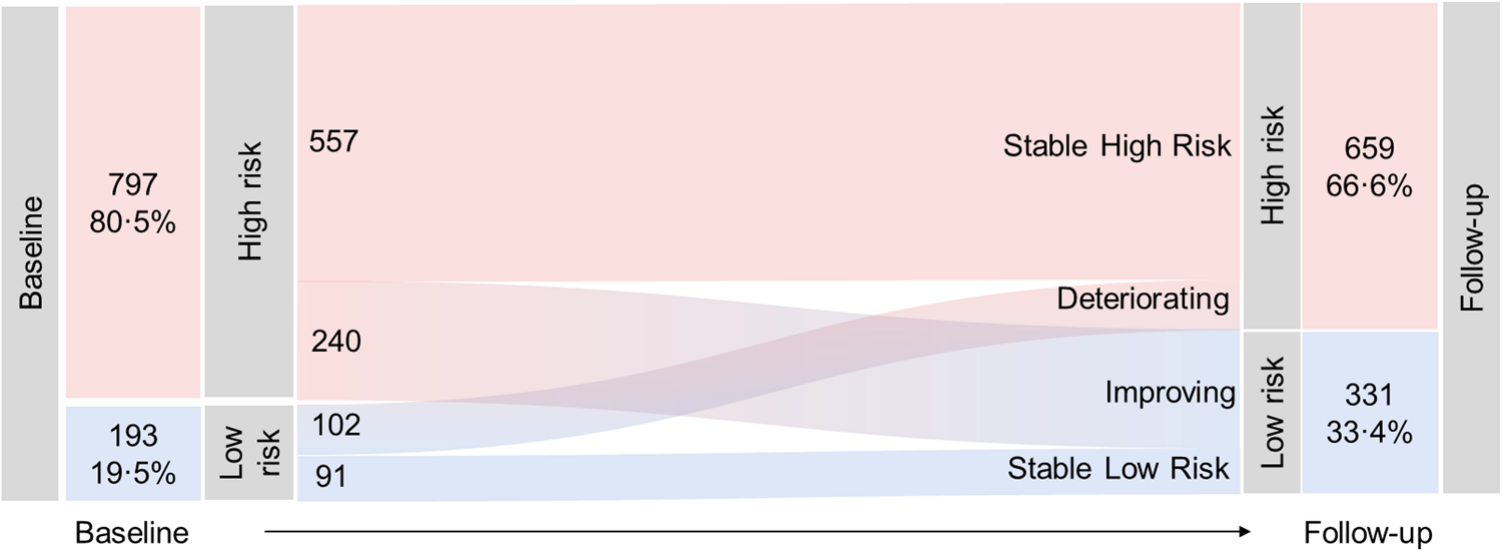
Mental health risk trajectories from baseline to follow-up. This illustrates the number of children in each of the four resilience trajectories. Red sections represent children at high risk (scoring above depression, PTSD, or externalising cutoffs) and blue sections represent children at low risk (below all cutoffs). *N* = 990 children with complete baseline and follow-up data

The majority of children had been exposed to war events (*n* = 1544, 96.9%). *N* = 297 (18.6%) war-exposed children fell below the cutoff for depression, PTSD, and externalising behaviour problems and were therefore classed as being resilient (defined as being at low risk for these mental health problems despite exposure to adversity). To identify predictors of resilience, these children were matched to children who fell above at least one cutoff (and hence were considered at risk of mental health problems) but who reported similar exposure to war events. The strongest predictor of being in the resilient group was self-esteem. The strongest predictors of being in the risk group were the temperament trait of environmental sensitivity, loneliness and social isolation, child maltreatment, caregiver PTSD, and caregiver depression. Results are discussed in Popham et al. (in revision). At follow-up, the proportion of children classed as resilient increased to 33.1%, with 8.7% of the cohort showing stable low risk for mental health problems and 24.4% showing significant improvement in mental health a year later (Fig. 2). However, 56.4% of children showed a stable high-risk trajectory and 10.5% had deteriorating mental health. Several aspects of the family environment, including social support, caregiver’s mental health, child–caregiver relationship, and child maltreatment, predicted children’s mental health over time. Some of these relationships were bidirectional: for example, child mental health symptoms were both predicted by and predictive of caregiver depression. This highlights the dynamic nature of risk and resilience and the complex interrelationships between children and the family environment, suggesting that systemic family-focused interventions may be appropriate. Results are discussed in Popham et al. (under review).

War exposure is a severe form of trauma that is associated with increased risk of mental health problems. Disruption of the hypothalamic–pituitary–adrenal axis, marked by disrupted cortisol secretion, may link the two. We explored the associations between hair cortisol concentrations (HCC), war exposure, and PTSD symptoms in children. War exposure was significantly associated with HCC, particularly in those who were aged 12 years or older at the time of exposure. HCC was also concurrently associated with PTSD symptoms, though it did not predict symptoms over time. These findings suggest that traumatic experiences can leave a lasting mark on the biology of the child, measurable as elevated HCC, particularly those in adolescence during exposure. Elevated HCC was indicative of greater burden of PTSD symptoms, though the nature of the relationship between them was unclear. The results are discussed in Smeeth et al. (under review).

## Strengths and limitations

There are several strengths to this cohort. The majority of refugees worldwide live in low- and middle-income countries, yet much of the research to date has been conducted with those who have moved to high-income countries; the focus on a cohort of Syrian refugees living in Lebanon close to the Syrian border helps to address this imbalance. Results should be generalisable to similar populations of Syrian refugees in the Middle East (i.e., those living in tented settlements), though care should be taken in extrapolating findings to those living in substantially different conditions (e.g. in urban areas) and those with different socioeconomic backgrounds.

Purposive cluster sampling was used to select a sample with varying levels of vulnerability, and a wide range of factors at the individual, family, and community levels were measured using both self-report and caregiver-report questionnaires per interview. Together with the inclusion of genetic, epigenetic, and neuroendocrine measures, this makes it possible to explore the interplay between biological and environmental factors in the development of mental health and wellbeing outcomes in children exposed to war and adversity associated with being a refugee. Furthermore, the study was designed to include children across a wide age range at baseline to make it possible to consider developmental aspects of mental health despite only two waves of data collection.

We also carried out clinical interviews in a representative subsample of the cohort to establish the validity of key outcome measures when used in this population and context. This provided validated cutoffs for psychopathology screening tools and allowed us to adjust estimated prevalence of mental disorders for false positives and false negatives.

We took several steps to reduce the risk of bias in the cohort. All eligible families from each ITS were invited to participate and one child was randomly selected from each family by the research team using a pre-specified approach. However, it is possible that older children were less likely to be included because they were working. Child-headed families and unaccompanied minors were not recruited because there was no adult to provide consent. Caregivers with more severe mental health problems may have been less likely to participate, and children with mental health problems that made establishing assent and participation challenging, such as selective mutism, were not included. Some settlements could not be visited for security reasons or because the settlement had been relocated out of the area. Collectively, these challenges are likely to have resulted in bias away from the most vulnerable children and families.

We were able to successfully follow up the target of 63% of the baseline sample. The follow-up sample was broadly representative of the baseline sample, though there were some differences. Those lost to follow-up were less likely to have access to education or be registered with UNHCR, and more likely to be from the most vulnerable localities, suggesting that the most vulnerable families were more difficult to follow up. However, differences between those retained versus not retained were mostly small (Table 1), suggesting that the follow- up sample was not substantially biased. We were also not able to follow up families who had moved to other areas of Lebanon or to other countries. This means we are likely to have missed some families who were subject to evictions as well as those with the means to move to other countries.

There are some limitations that should be considered. Only two waves of data are available, and due to the high mobility of the sample there are no plans to conduct further data collection. Self-report data is likely to be subject to bias, especially in younger children. For example, there is the possibility of recall bias in relation to war exposure and other risk and protective factors, which may be biased by the child’s current mental state and limited by some children’s very young age at the time of war exposure. However, the inclusion of both child and caregiver reports in several domains, as well as repeating measures at follow-up, will make it possible to explore bias in more detail. Measurement of child psychopathology relied on questionnaires, which have some limitations compared to clinical interviews. Reliability and validity of these measures was broadly sufficient and enabled adjustment of prevalence estimates, though the validity of the anxiety measure was less optimal [6]. More broadly, there are challenges associated with differentiating mental disorders from distress and behavioural responses that are understandable or potentially adaptive in the context of a refugee camp. These issues are explored in detail in Kyrillos et al. [9].

## Publications and availability of the data

Initial findings in relation to resilience, prevalence and predictors of mental health problems, and mental health assessment in this setting are available or will be available soon (Popham et al., in revision; Popham et al., under review; Kyrillos et al., in press; [9]), as well as the theoretical framework that will be used to explore the role of epigenetic factors [32]. More details about the study can be found at https://www.qmul.ac.uk/sbbs/about-us/our-departments/psychology/global-mental-health/. Researchers interested in collaboration should contact Professor Michael Pluess (m.pluess@qmul.ac.uk).

## Supplementary Information

Below is the link to the electronic supplementary material.

Supplementary file1 (DOCX 228 kb)

## Data Availability

Researchers interested in accessing data should contact Professor Michael Pluess at Queen Mary University of London, UK (e-mail: m.pluess@qmul.ac.uk).

## Code availability

Contact Professor Michael Pluess at Queen Mary University of London, UK (e-mail: m.pluess@qmul.ac.uk).
