# Supplementary material for: Cohort profile: biological pathways of risk and resilience in Syrian refugee children (BIOPATH)
Source: Soc Psychiatry Psychiatr Epidemiol. 2022 Jan 18;57(4):873–83. doi: 10.1007/s00127-022-02228-8 (PMC8960581; doi:10.1007/s00127-022-02228-8)
Supplement: Supplementary file 1 — Supplementary file1 (DOCX 228 kb) [file 127_2022_2228_MOESM1_ESM.docx]

**Supplementary materials**

**Contents**

[**Supplementary Section 1. Ethical approval** 2](#_Toc90229626)

[**Supplementary Section 2. Sampling and recruitment approach** 3](#_Toc90229627)

[**Supplementary Section 3. Child and caregiver age** 5](#_Toc90229628)

[**Supplementary Section 4. Time since leaving Syria** 6](#_Toc90229629)

[**Supplementary Section 5. Caregiver education and employment** 7](#_Toc90229630)

[**Supplementary Section 6. Clinical interview subsample** 8](#_Toc90229631)

[**Supplementary Section 7. Measures** 12](#_Toc90229632)

[**References** 23](#_Toc90229633)

# **Supplementary Section 1. Ethical approval**

Ethical approval was granted by the Institutional Review Board of the University of Balamand / Saint George Hospital University Medical Center, Lebanon (ref: IRB/O/024-16/1815). The study was also reviewed by the Lebanese National Consultative Committee on Ethics and approved by the Ministry of Public Health. The sponsor, Queen Mary University of London, reviewed the study for compliance with all relevant legal and regulatory requirements.

Mental health services were offered to anyone from participating communities; to reduce the risk of perceived pressure to participate, service access was not dependent on study participation. Services were provided by an international NGO that delivers primary care and mental health services in Lebanon, either as part of their standard services or through a clinical trial linked to the BIOPATH study (ClinicalTrials.gov ID: NCT03887312).

# **Supplementary Section 2. Sampling and recruitment approach**

Purposive cluster sampling was used, selecting seven municipalities with varying levels of vulnerability. The Lebanon Inter-Agency Coordination group defines five levels of vulnerability based on the Multi-Deprivation Index, Lebanese population dataset, and refugee population figures (1); it was possible to identify municipalities with sufficient ITSs across the most vulnerable to third most vulnerable levels. In these localities, small-to-medium sized ITS’s were selected and permission to access them was secured from the Ministry of Defense, local army intelligence units, and from municipalities where necessary. The community leader (chawich) of each ITS was approached to seek agreement to conduct the study. Recruitment took place on a subsequent day so that the community leader had time to inform residents of the ITS about the study. There was someone available to speak to the research team at 97.9% of shelters.

Families who were interested and eligible completed the informed consent procedure. Assent was taken from children only if their caregiver had consented to participation. The informed consent process was adjusted to account for low literacy and educational levels. The study was explained verbally by trained research staff, supported by a written information sheet and a simplified ‘easy read’ version of the information sheet with photos demonstrating sample collection. Research staff checked participants’ understanding of the study, addressed misunderstandings, answered questions and discussed concerns. Caregivers and children were asked to sign consent / assent forms or provide a thumb print in lieu of a signature. While financial compensation was offered to families for their time, research staff did not tell families about this until after they had agreed to take part.

Follow up was completed one year later. Each community leader was contacted for permission to re-visit the ITS. To maximise the chance of being able to re-contact families, we asked the community leader and several families in each ITS (contacted by phone) to inform other families of the visit date. If families had moved, information was taken over the phone or from neighbours about where they had moved to and their reason for moving (if known). Residents of some ITSs had moved due to evictions and it was not always possible to get information about where families had moved to. Participants were eligible for follow up if the same child and caregiver were available. If a child was no longer living with the same caregiver (typically because the child had married) the child was asked if there was another caregiver who knew them well enough to complete the survey; if not, they could opt to participate without a caregiver. In some cases, the caregiver differed because they had moved away or the primary caregiver had returned from abroad.

Data from six families were completely excluded at baseline for the following reasons: data missing because of tablet failure during data collection; child was <8 years old and clearly not able to understand questions; family was not from ITS; family participated twice. Data from eight families were excluded at follow up based on analysis of genetic data, primarily where there this confirmed that a different child had participated at follow up.


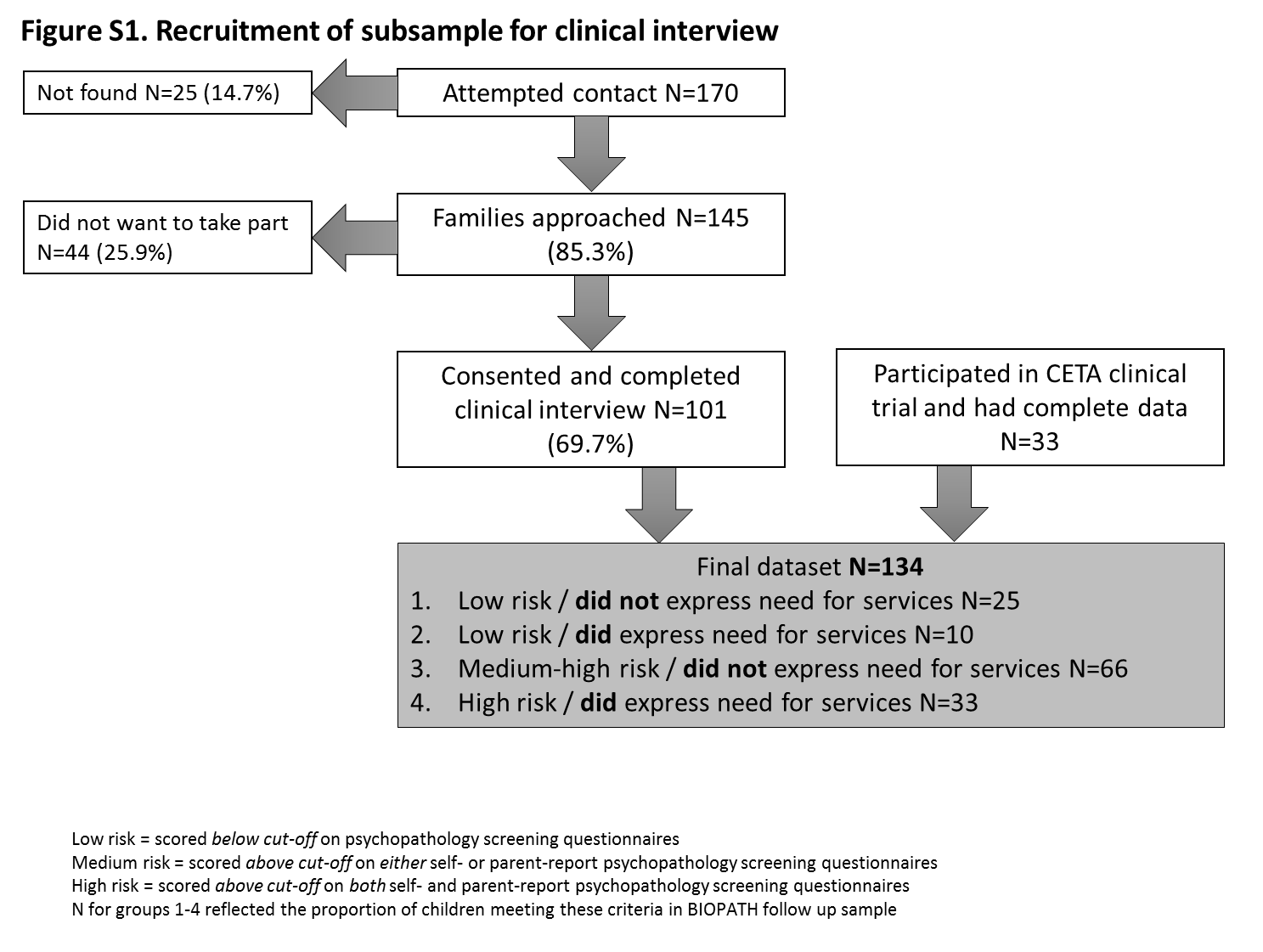


# **Supplementary Section 3. Child and caregiver age**

Eligibility criteria included child’s age between 8 and 16 years at recruitment; however, it was not uncommon for caregivers not to know or have a record of the child’s exact date of birth, hence in some cases there was some uncertainty about the child’s age. We did not exclude families who did not have documentation (e.g., UNHCR registration documents) so as not to bias the sample away from the most vulnerable families. At follow up, most families (96.3%) were registered with UNHCR and paperwork was checked to confirm the child’s identity. Date of birth was taken from UNHCR documentation where possible; however, it should be noted that UNHCR documents sometimes have an estimated date of birth if the individual did not have other documentation to present at registration.

Reported age and date of birth were recorded at each wave of data collection. Where both age and date of birth were available, data was inspected for consistency within and between waves (e.g., checking if reported age was consistent with date of birth and what was reported at baseline was consistent with follow up). Based on the consistency of data, the date of birth that was judged most likely to be correct was used to calculate the age at each data collection point and the degree of confidence in the calculated age was defined. In the majority of cases, we judged that we could be very confident (n=1092, 68.5%) or fairly confident (n=315, 19.8%) that the child’s age was correct. In a minority of cases (n=187, 11.7%) there was greater uncertainty about the exact age. Where date of birth was missing or clearly incorrect, only the reported age was used. The age variable reported in this paper is based on the calculated age rounded to the nearest year or, where date of birth was missing or not accurate, the reported age.

Using this new age variable the age range at recruitment was 6-19 years. While this means that some children fell outside of the age range we planned to sample, we did not automatically exclude the data from children on this basis. In one case the interviewers noted that the child seemed younger than 8 years and clearly struggled to understand the questions; this child’s data was excluded from analysis. Other children that were possibly younger than 8 years, but where the interviewer did not express concerns about their understanding, were flagged in the dataset. Where appropriate, analyses will be repeated excluding cases where it is possible that the child was younger than 8 years at baseline.

The same approach was taken with caregiver age: the age variable used in this paper is the calculated age rounded to the nearest year or, where date of birth was missing or not accurate, the reported age. In the majority of cases, we judged that we could be very confident (n=1164, 73.0%) or fairly confident (n=201, 12.6%) that the caregiver’s age was correct. In a minority of cases (n=229, 14.4%) there was greater uncertainty about the exact age.

# **Supplementary Section 4. Time since leaving Syria**

Caregivers reported when they left Syria at each wave of data collection as a time range (e.g., 0-6 months ago, 7-12 months ago, etc.). Given the date of data collection, the earliest and latest dates they could have left Syria were calculated for each wave. The earliest time category was unbounded (i.e. more than 4 years ago), therefore the start of the Syrian civil war was used as the earliest time point for this category (15^th^ March 2011). For those with two waves of data we calculated how overlapping the two reported date ranges were to give some indication of how consistent the data were between waves. To overcome any discrepancies in reported time between the two waves, we took the midpoint of each reported time range and took the average of the dates. This was then converted back into a categorical variable in 12 month bands.

There were n=591 (37.1%) families in which there were only data from baseline. In n=512 (32.1%) families there were data from baseline and one year follow up and the reported time ranges overlapped; in n=389 (24.4%) families the reported ranges did not overlap but the gap was less than one year; in n=103 (6.5%) cases the ranges did not overlap and the gap was greater than a year.

**Table S1. Reported time^A^ since leaving Syria, N (%)**

| Baseline | | 1 year follow up | |
| --- | --- | --- | --- |
| 0-12 months ago | 295 (18.5%) | 0-12 months ago | 4 (0.4%) |
| 12-24 months ago | 229 (14.4%) | 12-24 months ago | 160 (15.9%) |
| 24-36 months ago | 220 (13.8%) | 24-36 months ago | 126 (12.5%) |
| 36-48 months ago | 599 (37.6%) | 36-48 months ago | 179 (17.8%) |
| >48 months ago | 246 (15.4%) | 48-60 months ago | 292 (29.0%) |
|  |  | >60 months ago | 246 (24.4%) |
| Missing | 6 (0.4%) |  | 0 (0.0%) |
| Total | 1595 |  | 1007 |

^A^ Time was based on average of midpoint of ranges given at baseline and one year follow up if both were available; otherwise it was based on report at baseline

# **Supplementary Section 5. Caregiver education and employment**

**Table S2. Caregiver education and previous employment, N (%)**

|  | | Baseline | 1 year follow up |
| --- | --- | --- | --- |
| Highest level of education achieved by caregiver completing interview | Did not attend school | 900 (56.5%) | 562 (56.1%) |
|  | Basic educational certificate (up to grade 6) | 527 (33.0%) | 320 (32.0%) |
|  | General intermediate education (up to grade 9) | 130 (8.2%) | 79 (7.9%) |
|  | General secondary education certificate (up to grade 12) | 25 (1.6%) | 16 (1.6%) |
|  | Diploma or Bachelor degree | 7 (0.4%) | 4 (0.4%) |
|  | Higher diploma or Masters degree | 1 (0.1%) | 0 (0.0%) |
|  | Missing | 4 (0.3%) | 20 (2.0%) |
| Job of caregiver or spouse (highest earner) in Syria before the war | Manager or professional | 24 (1.5%) | 17 (1.7%) |
|  | Supervisor, clerical, skilled manual worker, service or sales | 512 (32.1%) | 301 (30.1%) |
|  | Elementary occupations, farming, construction | 869 (54.5%) | 530 (52.9%) |
|  | Never worked / long-term unemployed | 118 (7.4%) | 86 (8.6%) |
|  | Missing | 71 (4.5%) | 67 (6.7%) |

**Table S3. Can caregiver / adults in household read and write, N (%)**

|  | Baseline | 1 year follow up |
| --- | --- | --- |
| Not at all | 326 (20.4%) | 204 (20.4%) |
| A little | 579 (36.3%) | 319 (31.9%) |
| More or less | 340 (21.4%) | 213 (21.3%) |
| Mostly | 226 (14.2%) | 128 (12.8%) |
| Absolutely yes | 118 (7.4%) | 127 (12.7%) |
| Missing | 5 (0.3%) | 10 (1.0%) |

Caregivers’ highest level of education did not predict retention at follow up (χ^2^ (5)=1.99, p=.850), nor did the job of the highest earner pre-war (χ^2^ (3)=2.86, p=.414). However, households with the lowest literacy levels were less likely to be retained at follow up (χ^2^ (4)=10.34, p=.035, Somers’ d=.05).

# **Supplementary Section 6. Clinical interview subsample**

**Table S4. Comparison of clinical interview subsample to baseline sample**

|  | | Baseline (N=1594) | Clinical interview (N=134) | Comparison of those with clinical interview vs. those not included on baseline measures | |
| --- | --- | --- | --- | --- | --- |
|  |  |  |  | Test statistic | Details |
| Child gender, N (%) female | | 839 (52.6%) | 63 (47.0%) | χ^2^ (1)=1.85, p=.173, tau^A^=.001 |  |
| Child age at assessment, mean (SD) [range]^B^ | | 11.44 (2.44)  [6-19] | 12.15 (2.15)  [8-19] | t (164.08)=3.58, p<.001, d=0.30 | Included children are younger than those not included |
| Caregiver gender, N (%) female / male | | 1520 (95.4%) | 130 (97.0%)^C^ | χ^2^ (1)=3.28, p=.070, tau^A^=.002 |  |
| Caregiver age at assessment, mean (SD) [range]^B^ | | 39.03 (8.59) [18-75] | 38.07 (7.54) [16-57] | t (170.69)=3.40, p<.001, d=0.26 | Included children had younger caregivers |
| Caregiver relationship to child, N (%) | Mother | 1424 (89.3%) | 123 (91.8%)^C^ | χ^2^ (11)=11.99, p=.364, tau^A^=.005 |  |
|  | Father | 65 (4.1%) | 20 (14.9%)^C^ |  |  |
|  | Stepmother | 25 (1.6%) | 2 (1.5%) |  |  |
|  | Grandmother | 24 (1.5%) | 1 (0.7%) |  |  |
|  | Sister | 21 (1.3%) | 3 (2.2%) |  |  |
|  | Aunt | 18 (1.1%) | 0 (0.0%) |  |  |
|  | Brother | 6 (0.4%) | 0 (0.0%) |  |  |
|  | Uncle | 3 (0.2%) | 0 (0.0%) |  |  |
|  | Cousin | 3 (0.2%) | 0 (0.0%) |  |  |
|  | Other | 5 (0.3%) | 1 (0.7%) |  |  |
|  | Missing ^D^ | 0 (0.0%) | 0 (0.0%) |  |  |
|  |  |  |  |  |  |
|  |  |  |  |  |  |
| Time since leaving Syria (at baseline), N (%) | ≤3 years ago | 744 (46.7%) | N/A | χ^2^ (1)=2.52, p=.112, d^D^=.02 |  |
|  | >3 years ago | 844 (52.9%) |  |  |  |
|  | Missing | 6 (0.4%) |  |  |  |
| Child nationality, N (%) | Syrian | 1571 (98.6%) | N/A | χ^2^ (4)=1.77, p=.778, tau^C^=.001 |  |
|  | Lebanese | 8 (0.5%) |  |  |  |
|  | Palestinian | 13 (0.8%) |  |  |  |
|  | Iraqi | 1 (0.1%) |  |  |  |
|  | Missing | 1 (0.1%) |  |  |  |
| Caregiver nationality, N (%) | Syrian | 1577 (98.9%) | N/A | χ^2^ (3)=5.28, p=.152, tau^C^=.005 |  |
|  | Lebanese | 8 (0.5%) |  |  |  |
|  | Palestinian | 5 (0.3%) |  |  |  |
|  | Iraqi | 1 (0.1%) |  |  |  |
|  | Missing ^B^ | 3 (0.2%) |  |  |  |
| Child married / engaged, N (%) | | 26 (1.6%) | N/A | χ^2^ (1)=0.00, p=1.000, tau^C^=.00 |  |
| UNHCR vulnerability rating, N (%) | Most vulnerable | 615 (38.6%) | 114 (85.7%) | χ^2^ (2)=138.57, p<.001, d^D^=.13 | Included families more likely to be from most vulnerable localities |
|  | Second most vulnerable | 648 (40.6%) | 15 (11.3%) |  |  |
|  | Third most vulnerable | 332 (20.8%) | 4 (3.0%) |  |  |
| Family members registered with UNHCR, N (%) | All | 1253 (78.6%) | N/A | χ^2^ (2)=1.43, p=.490, d^D^=.000 |  |
|  | Some | 174 (10.9%) |  |  |  |
|  | None | 163 (10.2%) |  |  |  |
|  | Missing ^B^ | 4 (0.3%) |  |  |  |
| Number of people in household, median (IQR) [range] | Adults | 2 (1) [1-11] | N/A | U=87,637.0, p=.045 | Included families had fewer adults in the household |
|  | Children | 5 (2) [1-18] | N/A | U=103,574.0, p=.169 |  |
|  | Total | 7 (3) [2-24] | N/A | U=96,681.5, p=.906 |  |
| Caregiver has current job, N (%) | No | 1384 (86.8%) | N/A | χ^2^ (1)=0.82, p=.366, tau^C^=.001 |  |
|  | Yes | 206 (12.9%) |  |  |  |
| Child has access to education, N (%) | No school | 621 (39.0%) | N/A | χ^2^ (2)=32.95, p<.001, d^D^=.07 | Included children were more likely to have access to education |
|  | Some education | 398 (25.0%) |  |  |  |
|  | School | 573 (35.9%) |  |  |  |
|  | Missing | 2 (0.1%) |  |  |  |
| Highest level of education achieved by caregiver completing interview, N (%) | Did not attend school | 900 (56.5%) | N/A | χ^2^ (5)=3.16, p=.675, d^D^=.004 |  |
|  | Basic educational certificate (up to grade 6) | 527 (33.0%) |  |  |  |
|  | General intermediate education (up to grade 9) | 130 (8.2%) |  |  |  |
|  | General secondary education certificate (up to grade 12) | 25 (1.6%) |  |  |  |
|  | Diploma or Bachelor degree | 7 (0.4%) |  |  |  |
|  | Higher diploma or Masters degree | 1 (0.1%) |  |  |  |
|  | Missing | 4 (0.3%) |  |  |  |
|  |  |  |  |  |  |
|  |  |  |  |  |  |
| Job of caregiver or spouse (highest earner) in Syria before the war, N (%) | Manager or professional | 24 (1.5%) | N/A | χ^2^ (3)=1.33, p=.723, d^D^=.007 |  |
|  | Supervisor, clerical, skilled manual worker, service or sales | 512 (32.1%) |  |  |  |
|  | Elementary occupations, farming, construction | 869 (54.5%) |  |  |  |
|  | Never worked / long-term unemployed | 118 (7.4%) |  |  |  |
|  | Missing | 71 (4.5%) |  |  |  |
| Can adults in household read and write? N (%) | Not at all | 326 (20.4%) | N/A | χ^2^ (4)=1.89, p=.757, d^D^=.006 |  |
|  | A little | 579 (36.3%) |  |  |  |
|  | More or less | 340 (21.4%) |  |  |  |
|  | Mostly | 226 (14.2%) |  |  |  |
|  | Absolutely yes | 118 (7.4%) |  |  |  |
|  | Missing | 5 (0.3%) |  |  |  |

^A^ Goodman-Kruskal tau. ^B^ Age is best estimate rounded to nearest year, based on all date of birth and age data available (see Supplementary section 1); caregiver age is missing for n=3 cases. ^C^ In n=16 cases both parents attended the appointment; total for *Caregiver gender* and *Caregiver relationship to child* is n=150; in n=94 cases the clinical interview was completed with both child and caregiver(s) and in n=40 it was completed with the child only. ^D^ Somers’ d (if not marked, Cohen’s *d* is used). N/A, not asked at clinical interview appointment.

# **Supplementary Section 7. Measures**

**Table S4. Description of psychosocial measures**

| Domain | Child or caregiver report | Instrument | Description / modifications |
| --- | --- | --- | --- |
| Demographic and medical | | | |
| Demographic data | Both | Questions developed in collaboration with IDRAAC and NYU | Age, gender, caregiver relationship to child, nationality, religion, number of wives, consanguinity, region in Syria family from, time since leaving Syria, UNHCR registered, child married/engaged, parent deceased, child’s house chores and work, family/household size, income/assistance, caregiver working, caregiver education and former employment, household adult literacy |
| Medical data | Both | Questions developed in collaboration with IDRAAC | Child health (general health, recent illness/injury/dental problems, medication, smoking), caregiver health (general health, illness/disability), family health (illness/disability in other family members) |
| Puberty | Child | Pubertal Development Scale (PDS; abbreviated) (2) | Items with a sexual association (breast development, pubic hair growth) were omitted, leaving a two item scale for boys (facial hair, deepening of voice) and girls (menarche, age of onset) |
| Mental health service use for child, caregiver, and family members | Both | Mental Health Service Use and Needs (MhSUN) | Service use and needs reported for child, caregiver, and other family members; developed for this study |
| Perceived need for mental health services for child, caregiver, and family members | Both |  |  |
| Child mental health and wellbeing | | | |
| Wellbeing | Child | WHO-5 Well-Being Index (WHO-5) (3, 4) | Minor changes to translation to ensure comprehensibility |
| Post-traumatic stress disorder | Child | Child PTSD Symptom Scale (CPSS) (5) | Instructions supplemented to clarify the types of events probed for and timing of events; clarification added to one symptom item; functional impairment scale not used; items added to probe for past episodes |
| Depression | Child | Center for Epidemiological Studies Depression Scale for Children (CES-DC, abbreviated) (6-8) | Reduced to 10 items following piloting; items added to probe for past episodes |
| Anxiety | Child | Screen for Child Anxiety Related Emotional Disorders (SCARED, abbreviated) (9-11) | Reduced to 15 items for baseline; 3 items added back in so 18 items at follow up; removed items related to school, retaining subscales for panic disorder, generalised anxiety disorder, separation anxiety, social anxiety; items added to probe for past episodes |
| Nightmares | Caregiver | Children’s Sleep Habits Questionnaire (CSHQ, abbreviated) (12) | Three items measuring aspects of trauma-related sleep symptoms selected from scale |
| Night screams | Caregiver |  |  |
| Bedwetting | Caregiver |  |  |
| Externalising behaviour problems | Caregiver | Strengths and Difficulties Questionnaire (SDQ): Externalising score (13-15) | SDQ administered in its entirety as published; conduct and hyperactivity subscales used as measure of externalising behaviour problems |
|  |  | Items aligned with DSM-5 conduct disorder and oppositional defiant disorder criteria (16) | 12 items developed for this study, omitting sensitive areas (forced sexual activity) and more severe behaviours (e.g., firesetting, use of a gun) |
| Externalising behaviour problems (cont.) | Caregiver | Items aligned with DSM-5 attention deficit hyperactivity disorder (16) | 6 items developed by IDRAAC |
| Impairment / disability | Caregiver | Strengths and Difficulties Questionnaire (SDQ) Impact supplement (17) | SDQ administered in its entirety as published; impact supplement used as measure of impairment due to mental health problems |
|  | Both | Adapted World Health Organization Disability Assessment Schedule (WHO DAS) (18-21) | 24-item adapted scale with Global Disability score and subscales: Getting along with others, Life activities, Participation in society, Activity limitation days, Overall health, Overall impairment |
| Psychopathology | Both | Mini International Neuropsychiatric Interview for Children and Adolescents (MINI Kid) v6.0; additional questions to assess against DSM-5 criteria; Clinical Global Impression – severity (CGI-s) score (22-24) | Under clinical supervision, some adaptations were made to account for culturally sensitive issues and assessing problem behaviours in the refugee context; further questions to elicit information to assess symptoms against DSM-5 criteria were developed by an experienced clinical psychologist; consensus diagnosis was reached following supervision and based on information from the clinical interview, CGI-s score, and expert clinical opinion |
| Individual level associates / predictors | | | |
| Optimism | Child | Youth Life Orientation Test (YLOT, abbreviated) (25) | Four optimism items were selected, dropping two that might be less indicative of optimism in a refugee setting |
| Self-esteem | Child | Lifespan Self-Esteem Scale (LSE, abbreviated) (26) | Selected one item following piloting with Syrian refugee children in Lebanon (all 4 original items were perceived to mean the same) |
|  |  |  |  |
| Future aspiration | Child | Future Aspirations and Plans (FAP); measure of future orientation developed for this study | Developed based on focus groups with Syrian refugee children in Lebanon; adapted some items from Consideration of Future Consequences Scale (27) |
| Future expectation | Child |  |  |
| Future planning and motivation | Child |  |  |
| Self-efficacy | Child | General Self-Efficacy Scale (GSE 4) (28, 29) | Selected 4 items most suitable for children; replaced one following piloting |
| Problem-focused coping | Child | Children’s Coping Strategies Checklist (CCSC, abbreviated) (30, 31) | Reduced to 15 items, 3 per subscale, based on pilot testing with Syrian children in Lebanon |
| Cognitive restructuring coping | Child |  |  |
| Distraction coping | Child |  |  |
| Avoidance coping | Child |  |  |
| Support-seeking coping | Child |  |  |
| Environmental sensitivity | Child | Highly Sensitive Child Scale (HSC) (32) | 12 item version of scale used; modified 3 items following pilot testing to reduce possible social desirability effects; developed visual scale to aid use of the 7-point Likert scale response format |
| Child religiosity | Child | Items on religious activity, orientation, and coping from NICHD Study of Early Child Care and Youth Development, Barber et al., National Study of Youth and Religion Survey, IDRAAC (33-35) | After piloting with Syrian children in Lebanon, removed an item on attending religious meetings to avoid gender bias; adjusted response options for some items to reduce ceiling effects |
| Family level associates / predictors | | | |
| Child maltreatment (child & caregiver reported) | Both | ISPCAN Child Abuse Screening Tool (ICAST, abbreviated) (36) | Shortened to 22 items for children and 9 items for caregivers; some items clarified; sexual abuse items removed due to sensitivity and replaced by item enquiring about “private events” (the child was not required to disclose the details); after completing the ICAST, child was asked if they wanted to talk to a case manager about related issues and referrals made as necessary |
| Forced marriage | Child | Child asked if anyone had tried to force them into marriage | Developed for this study |
| Psychological control (disrespect): mother ^A^ | Child | Psychological Control – Disrespect Scale (PCDS) (37) | No modifications, previously adapted for similar context |
| Psychological control (disrespect): father ^A^ | Child |  |  |
| Acceptance: mother ^A^ | Child | Acceptance subscale – Child report of Parent Behavior Inventory (CRPBI) (38) | No modifications, previously adapted for similar context |
| Acceptance: father ^A^ | Child |  |  |
| Parental monitoring | Child | Parental Monitoring Scale (PMS) (39) | No modifications, previously adapted for similar context |
| Parent-child conflict | Child | Parent-Adolescent Conflict (40) | Response options were reduced from 6 to 5 and reworded to be consistent with items on positive home experiences (below) |
| Positive home experiences | Child | Positive Home Experiences (PHE, abbreviated) | Four items asking about presence of parents during day to day activities were used |
| Availability of toys | Child | One question asking whether child had toys to play with | Developed for this study |
| Caregiver wellbeing | Caregiver | WHO-5 Well-Being Index (WHO-5) (3, 4) | Minor changes to translation to ensure comprehensibility |
| Caregiver self-efficacy | Caregiver | General Self-Efficacy Scale (GSE, abbreviated) (28, 29) | Selected 4 items most suitable for children; replaced one following piloting |
| Caregiver environmental sensitivity | Caregiver | Highly Sensitive Person Scale – Brief Version (HSP-12) (41) | Modified 1 item to aid comprehensibility in Arabic; developed visual scale to aid use of the 7-point Likert scale response format |
| Life events | Caregiver | Life Events (42) | 7-point Likert scale converted to two stage question for each event: (i) Did the event affect them in a positive, negative, or neutral way; (ii) Did the event affect them only a little, quite a lot, a great deal? |
| Caregiver religiosity | Caregiver | Items on religious activity, orientation, and coping from NICHD Study of Early Child Care and Youth Development, Barber et al., National Study of Youth and Religion Survey, IDRAAC (33-35) | Adjusted response options for some items to reduce ceiling effects |
| Caregiver PTSD | Caregiver | PTSD Checklist for DSM-5 (PCL-5) (43) | Items added to probe for past episodes in self, child, and child’s father |
| Caregiver anxiety | Caregiver | Depression Anxiety and Stress Scale (DASS-21): Anxiety subscale (44, 45) | Items added to probe for past episodes in self, child, and child’s father |
| Caregiver depression | Caregiver | Center for Epidemiologic Studies Short Depression Scale (CES-D 10) (46) | Items added to probe for past episodes in self, child, and child’s father |
| Caregiver impulsivity | Caregiver | Abbreviated Barratt Impulsiveness Scale (ABIS): Attentional, Motor, Non-planning scales (47) | Removed one item that was not well understood during piloting with Syrian refugees in Lebanon |
| Caregiver general mental health | Caregiver | Single-item measures of self-rated mental health (SRMH); current, during war, pre-war (48) | Three items developed to cover: (i) past year; (ii) since war started but prior to past year; (iii) prior to the war |
| Caregiver perceived stress | Caregiver | Perceived Stress Scale (PSS-4) (49) | Removed items that were difficult to interpret in refugee context and replaced with similar items from PSS-10 |
| Livelihood | Caregiver | Perceived Refugee Environment Index (PREI) | Developed for this study as multidimensional measure to assess the quality of the refugee environment (see below for further subscales) |
| Basic needs | Caregiver |  |  |
| Housing | Caregiver |  |  |
| Family environment | Caregiver |  |  |
| Learning environment | Caregiver |  |  |
| Community level associates / predictors | | | |
| War exposure | Both | War Events Questionnaire (WEQ) (50) | Checklist of 19 war events completed by child and caregiver (reporting on child’s exposure to events); 6 events are two-part questions resulting in 25 item checklist |
| Bullying | Child | Bullying of Refugee Children (BRC) | Developed for this study based on advice from agencies working with Syrian refugee children in Lebanon about types of victimisation commonly experienced and modified following piloting: 8 items included at baseline and 12 items at follow up |
| Forced work | Child | Three items about forced work | Developed for this study |
| Child maltreatment outside the home | Child | Five items were adapted from ICAST to ask about maltreatment by adults outside of the home and family | Developed for this study |
| Loneliness and social isolation | Child | Loneliness in Refugee Children (LRC) (51) | Two items adapted from Loneliness and Social Dissatisfaction Scale Items; two items were written to capture social isolation related to refugee context |
| Perceived social support ^B^ | Child | Multidimensional Scale of Perceived Social Support for Arab American Adolescents (MSPSS-AA): family and friends subscales (52) | Subscales for social support from family and friends used with all children (see below for subscale for social support from teachers); used 5-point rating scale instead of 3-point scale |
| Perceived security | Child | Positive Home Experiences (PHE) | Two items asking about child’s perception of security were used |
| Access to education | Both | Child asked if they attend school; caregiver rated extent to which child had access to basic education / when they last attended school | Information from child and parent report were used to rate access to education on three point scale |
| Peer victimisation at school ^C^ | Child | Perceptions of Peer Social Support Scale (PPSSS; NICHD version, abbreviated) (53, 54) | Two items were used (a further two items that did not perform well in piloting were removed); one item amended for clarity |
| Peer support at school ^C^ | Child |  | Five items selected to assess social support |
| Teacher support at school ^C^ | Child | Multidimensional Scale of Perceived Social Support for Arab American Adolescents (MSPSS-AA): teacher subscale (52) | Subscale for social support from teachers used in those children who reported attending school (see above for subscales related to family and friends); removed the term counsellor from items; used 5-point rating scale instead of 3-point scale |
| School connectedness ^C^ | Child | School Connectedness (55) | Some rephrasing of items for consistency with other school measures |
| Access to services | Caregiver | Perceived Refugee Environment Index (PREI) | Developed for this study as multidimensional measure to assess the quality of the refugee environment (see above for further subscales) |
| Community environment | Caregiver |  |  |
| Working situation | Caregiver |  |  |
| Future mobility | Caregiver |  |  |
| Perceived refugee environment | Caregiver |  |  |
| Human insecurity | Caregiver | Human Insecurity Scale (HIS) (56) | No modifications, previously adapted for similar context |
| Collective efficacy | Caregiver | Collective Efficacy: Social Cohesion and Trust & Informal Social Control (57) | At follow up, asked extent to which neighbourhood had changed; added instructions to highlight change in direction of items that are scored negatively rather than positively |

^A^ If the mother was not regularly caring for the child, this was the female caregiver most involved in their care; if the father was not present in the home, this either referred to the father when they were last together or another male caregiver most involved in the child’s care (children could opt not to answer questions about father/male caregiver); ^B^ Perceived social support was assessed for both family and peers, so subscales cover both family and community level; ^C^ Completed by the subset of children who reported attending school

There were some data where there was a degree of uncertainty about validity, especially questions that had implications for resource allocation or were sensitive for other reasons. For example, there were sometimes discrepancies between child and caregiver report on whether the child’s father was in Lebanon. It is also not clear whether reports of income or work were accurate (refugees are typically not allowed to work). Some questions, for example about child maltreatment and caregiver-child relationship, may have been biased by children’s concerns about people overhearing them, and responses to questions about religious practice were likely biased by the importance of being seen to adhere to religious teachings. We used comprehensive feedback from interviewers to highlight these issues and guide interpretation of data. Furthermore, we repeated key demographic questions at each stage of data collection and used data from the two waves to identify and resolve discrepancies.

**
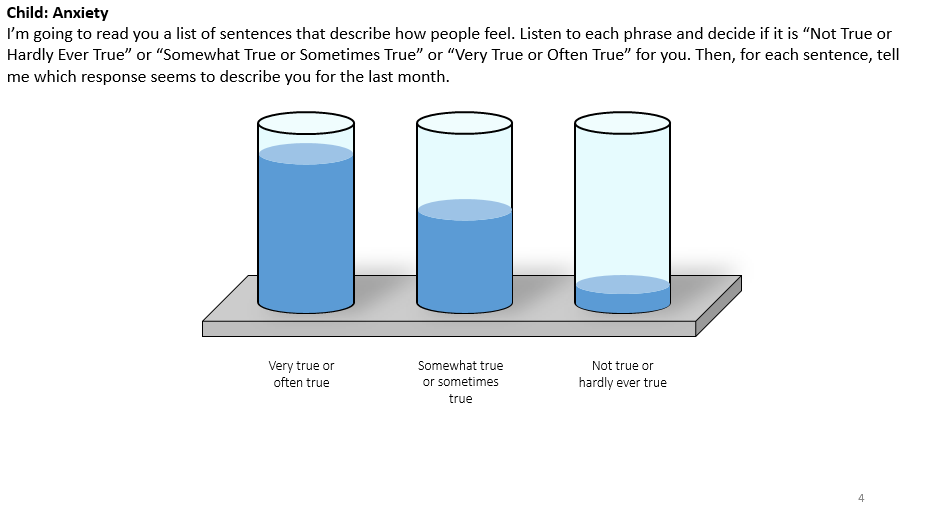
**

**Figure S2. Visual aid for use with the SCARED**

The versions used in the study were in Arabic, with glass order moving from right to left with the response options. This is one example from the SCARED; similar visual aids were used with each measure

# **References**

1. UNICEF. Equity in Crisis Response InterAgency Mapping Partners IAMP V33 Refugees in Informal Settlements Inter-Agency Coordination Lebanon December 2016 2017 [Available from: <https://www.refworld.org/docid/588748e84.html>.

2. Carskadon MA, Acebo C. A self-administered rating scale for pubertal development. J Adolesc Health. 1993;14(3):190-5.

3. Bech P. Clinical Psychometrics. Oxford: Wiley Blackwell; 2012.

4. Topp CW, Ostergaard SD, Sondergaard S, Bech P. The WHO-5 Well-Being Index: A Systematic Review of the Literature. Psychother Psychosom. 2015;84(3).

5. Foa EB, Johnson KM, Feeny NC, Treadwell KR. The Child PTSD Symptom Scale: A preliminary examination of its psychometric properties. Journal of clinical child psychology. 2001;30(3):376-84.

6. Weissman MM, Orvaschel H, Padian N. Children's symptom and social functioning self-report scales. Comparison of mothers' and children's reports. J Nerv Ment Dis. 1980;168(12):736-40.

7. Faulstich ME, Carey MP, Ruggiero L, Enyart P, Gresham F. Assessment of depression in childhood and adolescence: an evaluation of the Center for Epidemiological Studies Depression Scale for Children (CES-DC). Am J Psychiatry. 1986;143(8):1024-7.

8. Ayyash-Abdo H, Nohra J, Okawa S, Sasagawa S. Depressive Symptoms among Adolescents in Lebanon: A Confirmatory Factor Analytic Study of the Center for Epidemiological Studies Depression for Children. Acta Psychopathologica. 2016;2(6).

9. Birmaher B, Khetarpal S, Brent D, Cully M, Balach L, Kaufman J, et al. The screen for child anxiety related emotional disorders (SCARED): Scale construction and psychometric characteristics. Journal of the American Academy of Child and Adolescent Psychiatry. 1997;36(4):545-53.

10. Birmaher B, Brent DA, Chiappetta L, Bridge J, Monga S, Baugher M. Psychometric properties of the Screen for Child Anxiety Related Emotional Disorders (SCARED): a replication study. J Am Acad Child Adolesc Psychiatry. 1999;38(10):1230-6.

11. Hariz N, Bawab S, Atwi M, Tavitian L, Zeinoun P, Khani M, et al. Reliability and validity of the Arabic Screen for Child Anxiety Related Emotional Disorders (SCARED) in a clinical sample. Psychiatry Res. 2013;209(2):222-8.

12. Owens JA, Spirito A, McGuinn M. The Children's Sleep Habits Questionnaire (CSHQ): psychometric properties of a survey instrument for school-aged children. Sleep. 2000;23(8):1043-51.

13. Goodman R. The Strengths and Difficulties Questionnaire: A research note. Journal of Child Psychology and Psychiatry. 1997;38:581-6.

14. Goodman A, Lamping DL, Ploubidis GB. When to use broader internalising and externalising subscales instead of the hypothesised five subscales on the Strengths and Difficulties Questionnaire (SDQ): data from British parents, teachers and children. J Abnorm Child Psychol. 2010;38(8):1179-91.

15. Alyahri A, Goodman R. Validation of the Arabic Strengths and Difficulties Questionnaire and the Development and Well-Being Assessment. East Mediterr Health J. 2006;12 Suppl 2:S138-46.

16. APA. Diagnostic and Statistical Manual of Mental Disorders, Fifth Edition. 5th ed. Arlington, VA: American Psychiatric Association; 2013.

17. Goodman R. The extended version of the Strengths and Difficulties Questionnaire as a guide to child psychiatric caseness and consequent burden. J Child Psychol Psychiatry. 1999;40(5):791-9.

18. Betancourt T, Scorza P, Meyers-Ohki S, Mushashi C, Kayiteshonga Y, Binagwaho A, et al. Validating the Center for Epidemiological Studies Depression Scale for Children in Rwanda. J Am Acad Child Adolesc Psychiatry. 2012;51(12):1284-92.

19. Betancourt T, Scorza P, Kanyanganzi F, Fawzi MC, Sezibera V, Cyamatare F, et al. HIV and child mental health: a case-control study in Rwanda. Pediatrics. 2014;134(2):e464-72.

20. Betancourt TS, Ng LC, Kirk CM, Brennan RT, Beardslee WR, Stulac S, et al. Family-based promotion of mental health in children affected by HIV: a pilot randomized controlled trial. J Child Psychol Psychiatry. 2017;58(8):922-30.

21. Scorza P, Stevenson A, Canino G, Mushashi C, Kanyanganzi F, Munyanah M, et al. Validation of the "World Health Organization Disability Assessment Schedule for children, WHODAS-Child" in Rwanda. PLoS One. 2013;8(3):e57725.

22. Sheehan DV, Sheehan KH, Shytle RD, Janavs J, Bannon Y, Rogers JE, et al. Reliability and validity of the Mini International Neuropsychiatric Interview for Children and Adolescents (MINI-KID). J Clin Psychiatry. 2010;71(3):313-26.

23. Busner J, Targum SD. The clinical global impressions scale: applying a research tool in clinical practice. Psychiatry (Edgmont). 2007;4(7):28-37.

24. McEwen FS, Moghames P, Bosqui T, Kyrillos V, Chehade N, Saad S, et al. Validating screening questionnaires for internalising and externalising disorders against clinical interviews in 8-17 year-old Syrian refugee children. London, UK: Queen Mary University of London; 2020 2020.

25. Ey S, Hadley W, Allen DN, Palmer S, Klosky J, Deptula D, et al. A new measure of children's optimism and pessimism: The youth life orientation test. Journal of Child Psychology and Psychiatry. 2005;46(5):548-58.

26. Harris MA, Donnellan MB, Trzesniewski KH. The Lifespan Self-Esteem Scale: Initial Validation of a New Measure of Global Self-Esteem. J Pers Assess. 2018;100(1):84-95.

27. Strathman A, Gleicher F, Boninger DS, Edwards CS. The Consideration of Future Consequences: Weighing Immediate and Distant Outcomes of Behavior. Journal of Personality and Social Psychology. 1994;66(4):742-52.

28. Schwarzer R, Jerusalem M. Generalized Self-Efficacy scale. In: Weinman J, Wright S, Johnston M, editors. Measures in health psychology: A user’s portfolio Causal and control beliefs. Windsor, England: NFER-NELSON; 1995. p. 35-7.

29. Scholz U, Doña BG, Sud S, Schwarzer R. Is General Self-Efficacy a Universal Construct? Psychometric Findings from 25 Countries. European Journal of Psychological Assessment. 2002;18(3):242-51.

30. Program-for-Prevention-Research. Manual for the Children's Coping Strategies Checklist & How I Coped Under Pressure Scale. Manual. Tempe, AZ: Arizona State University; 1999.

31. Sandler IN, Tein JY, Mehta P, Wolchik S, Ayers T. Coping efficacy and psychological problems of children of divorce. Child Dev. 2000;71(4):1099-118.

32. Pluess M, Assary E, Lionetti F, Lester KJ, Krapohl E, Aron E, et al. Environmental Sensitivity in Children: Development of the Highly Sensitive Child Scale and Identification of Sensitivity Groups Developmental Psychology. 2018;54(1):51-70.

33. Barber BK. Political violence, social integration, and youth functioning: Palestinian youth from the Intifada. Journal of Community Psychology. 2001;29(3):259-80.

34. Pearce LD, Foster EM, Hardie JH. A Person-Centered Examination of Adolescent Religiosity Using Latent Class Analysis. J Sci Study Relig. 2013;52(1):57-79.

35. Denton ML, Pearce LD, Smith C. Religion and Spirituality On the Path Through Adolescence, Research Report Number 8: University of North Carolina at Chapel Hill; 2008.

36. Runyan DK, Dunne MP, Zolotor AJ. Introduction to the development of the ISPCAN child abuse screening tools. Child Abuse Negl. 2009;33(11):842-5.

37. Barber BK, Xia M, Olsen JA, McNeely CA, Bose K. Feeling disrespected by parents: refining the measurement and understanding of psychological control. J Adolesc. 2012;35(2):273-87.

38. Schaefer ES. Children's Reports of Parental Behavior: An Inventory. Child Dev. 1965;36:413-24.

39. Brown BB, Mounts N, Lamborn SD, Steinberg L. Parenting practices and peer group affiliation in adolescence. Child Dev. 1993;64(2):467-82.

40. Barber BK. Political violence, family relations, and Palestinian youth functioning. Journal of Adolescent Research. 1999;14(2):206-30.

41. Pluess M, Lionetti F, Aron EN, Aron A. People Differ in their Sensitivity to the Environment: An Integrated Theory and Empirical Evidence. PsyArXiv Preprints. 2020.

42. Taylor SE, Way BM, Welch WT, Hilmert CJ, Lehman BJ, Eisenberger NI. Early family environment, current adversity, the serotonin transporter promoter polymorphism, and depressive symptomatology. Biol Psychiatry. 2006;60(7):671-6.

43. Blevins CA, Weathers FW, Davis MT, Witte TK, Domino JL. The Posttraumatic Stress Disorder Checklist for DSM-5 (PCL-5): Development and Initial Psychometric Evaluation. J Trauma Stress. 2015;28(6):489-98.

44. Henry JD, Crawford JR. The short-form version of the Depression Anxiety Stress Scales (DASS-21): construct validity and normative data in a large non-clinical sample. Br J Clin Psychol. 2005;44(Pt 2):227-39.

45. Kulsoom B, Afsar NA. Stress, anxiety, and depression among medical students in a multiethnic setting. Neuropsychiatr Dis Treat. 2015;11:1713-22.

46. Radloff LS. The CES-D Scale: A self-report depression scale for research in the general population. Applied Psychological Measurement. 1977;1(3):385-401.

47. Coutlee CG, Politzer CS, Hoyle RH, Huettel SA. An Abbreviated Impulsiveness Scale constructed through confirmatory factor analysis of the Barratt Impulsiveness Scale Version 11. Archives of Scientific Psychology. 2014;2(1):1.

48. Kessler RC, Ustun TB. The World Mental Health (WMH) Survey Initiative Version of the World Health Organization (WHO) Composite International Diagnostic Interview (CIDI). Int J Methods Psychiatr Res. 2004;13(2):93-121.

49. Cohen S, Kamarck T, Mermelstein R. A global measure of perceived stress. Journal of Health and Social Behavior. 1983;24(4):385-96.

50. Karam EG, Al-Atrash R, Saliba S, Melhem N, Howard D. The War Events Questionnaire. Social Psychiatry and Psychiatric Epidemiology. 1999;34(5):265-74.

51. Asher SR, Hymel S, Renshaw PD. Loneliness in children. Child Development. 1984;55(4):1456-64.

52. Ramaswamy V, Aroian KJ, Templin T. Adaptation and psychometric evaluation of the multidimensional scale of perceived social support for Arab American adolescents. Am J Community Psychol. 2009;43(1-2):49-56.

53. Kochenderfer BJ, Ladd GW. Peer victimization: cause or consequence of school maladjustment? Child Dev. 1996;67(4):1305-17.

54. Ladd GW, Kochenderfer BJ, Coleman CC. Friendship quality as a predictor of young children's early school adjustment. Child Development. 1996;67(3):1103-18.

55. Resnick MD, Bearman PS, Blum RW, Bauman KE, Harris KM, Jones J, et al. Protecting adolescents from harm. Findings from the National Longitudinal Study on Adolescent Health. The Journal of the American Medical Association. 1997;278(10):823-32.

56. Ziadni M, Hammoudeh W, Rmeileh NM, Hogan D, Shannon H, Giacaman R. Sources of Human Insecurity in Post-War Situations: The Case of Gaza. J Hum Secur. 2011;7(3).

57. Sampson RJ, Raudenbush SW, Earls F. Neighborhoods and violent crime: A multilevel study of collective efficacy. Science. 1997;277(5328):918-24.
